# No Association between the rs1799836 Polymorphism of the Monoamine Oxidase B Gene and the Risk of Autism Spectrum Disorders in the Kazakhstani Population

**DOI:** 10.1155/2019/2846394

**Published:** 2019-06-02

**Authors:** Anastassiya V. Perfilyeva, Kira B. Bespalova, Liliya A. Skvortsova, Assel Surdeanu, Aleksandr A. Garshin, Yuliya V. Perfilyeva, Ozada Kh. Khamdiyeva, Bakhytzhan O. Bekmanov, Leyla B. Djansugurova

**Affiliations:** ^1^Institute of General Genetics and Cytology CS MES RK, Almaty 050000, Kazakhstan; ^2^Al-Farabi KazNU, Almaty 050000, Kazakhstan; ^3^M. Aitkozhin Institute of Molecular Biology and Biochemistry, Almaty 050000, Kazakhstan

## Abstract

Autism spectrum disorders (ASDs) are heterogeneous diseases that are triggered by a number of environmental and genetic factors. The aim of the current study was to investigate an association of the rs1799836 genetic variant of the neurotransmitter-related gene MAOB with ASDs. In total, 262 patients diagnosed with ASDs and their 126 healthy siblings were included in the present study. All individuals represented a Kazakhstani population. The distributions of the rs1799836 genotype were in accordance with the Hardy-Weinberg equilibrium among both cases and controls. No statistically significant differences were found in the allelic distributions of this polymorphism between ASD and control subjects (A/G: for males OR = 1.11, 95% 0.59-2.06, *p* = 0.75; for females OR = 1.14, 95% 0.70-1.86, *p* = 0.76). However, the increased score in the overall CARS was significantly associated with the A allele of rs1799836 MAOB for females (OR = 2.31, 95% 1.06-5.04, *p* = 0.03). The obtained results suggest that the rs1799836 polymorphism of the MAOB gene may have little contribution to the development of ASDs but may be involved in pathways contributing to ASD symptom severity in females. Further large-scale investigations are required to uncover possible relationships between rs1799836 MAOB and ASD progression in a gender-specific manner and their possible application as a therapeutic target.

## 1. Introduction

Autism spectrum disorders (ASDs) are complex childhood neuropsychiatric disorders mainly characterized by deficits in verbal and nonverbal communication, reciprocal social interactions, and stereotypical behaviors. In the last five years, the number of reported cases of children with autism in Kazakhstan has increased by 1.8 times. According to the official data provided by the Ministry of Healthcare of the Republic of Kazakhstan for 2018, the prevalence of ASD is 2.6 per 100,000 children, which is lower than the global statistics. WHO estimated that worldwide, 1 in 160 children has an ASD with an incidence rate of 6.25 per 100,000 people. We assume that the official data do not reflect the true picture of morbidity due to the difficulties of ASD diagnostics in our country.

The etiology of this pathology is extremely complex, and in most cases, the underlying pathological mechanisms remain unknown. In recent years, a number of works have been devoted to the study of neurochemical, immunological, structural, functional, and genetic factors of the ASD etiopathogenesis. According to the prevailing view, autistic disorders are pathophysiological processes caused by a combination of various exogenous factors encountered in the early stages of development, as well as genetic factors. Identification of candidate genes associated with a genetic predisposition to ASDs is a common molecular strategy [1].

In this study, we focused on the monoamine oxidase B (*MAOB*) gene, which has been considered as one of the candidate genes by a number of studies. Monoamine oxidase (MAO) is one of the most important enzymes in neurotransmitter metabolism. The MAOB is a member of the monoamine oxidase family of flavoproteins, which catalyze the oxidation of primary and secondary amines, polyamines, amino acids, and methylated lysine side chains in proteins [2]. MAOB is located in the outer mitochondrial membrane and expressed mainly in platelets, lymphocytes, and the brain [3], where it preferentially oxidizes benzylamine, beta-phenylethylamine (PEA), and dopamine [2].

PEA is a biogenic trace amine, which acts as a central nervous system stimulant. The role of PEA is not fully clear, but its chemical similarity to d-amphetamine suggests that this molecule facilitates catecholamine and serotonin release [4]. In the brain, PEA affects the mood and emotions and is able to increase mental focus. Abnormal concentrations of PEA may be associated with a number of psychological disorders [5]. It was shown that PEA, which is formed from phenylalanine, and phenylalanine itself are reduced in the urine of ASD patients [6].

Another target of the MAOB protein is dopamine, which plays an important role in the motor control and executive functions and in the regulation of arousal, motivation, reinforcement, and reward behaviors. It has been shown that ASDs are associated with dopaminergic dysfunctions [7–11]. Abnormal dopamine was demonstrated in the urine of autistic children through the measurement of the level of homovanillic acid as a degradation product of dopamine [12]. It has been shown that *de novo* mutations in the dopamine transporter gene lead to dopamine dysfunctions and may confer risk of ASDs [13]. Moreover, low maternal serum dopamine B hydroxilase levels have been demonstrated to create a nonideal intrauterine environment, which can lead to autistic disorders in the fetus [14].

In addition, it was shown that 30–50% of ASD patients have increased levels of blood serotonin (5-hydroxytryptamine (5-HT)) [15, 16], which plays a key role in the neuronal maturation, dendritic formation, and synaptogenesis in early developmental stages. In the condition of absence of MAOA, which is more affined for serotonin, the overlapping substrate specificities of the two MAO isoenzymes allow MAOB to participate in the metabolism of serotonin [17]. Taking into account that MAOB is the only isoenzyme expressed in platelets, it may play an important role in the regulation of plasma 5-HT levels. Moreover, male MAO A/B knockout mice exhibited a number of autistic-like behavioral changes, such as social and communication impairments, perseverative and stereotypical responses, and behavioral inflexibility, which were probably caused by high 5-HT levels in early developmental stages [18].

Aside from the important enzymatic functions, another possible reason to consider MAOB as a candidate gene for ASD susceptibility is its location on the X chromosome (Xp11.3). It is well known that ASDs are prevalent in males (4 males : 1 female) [1], indicating that ASD risk genes can be found in the X/Y chromosomes.

Genetic variations in the *MAOB* gene can affect its functions, contributing to the genetic predisposition to neurodegenerative diseases including ASDs. Therefore, in the current family-based study, samples from 262 Kazakhstani ASD patients and their 126 healthy brothers and sisters were analyzed for a single nucleotide polymorphism (SNP) in intron 13 of the *MAOB* gene (rs1799836), which results in the transitional conversion of adenine (A) to guanine (G) at the position 36 bp upstream from the start position of exon 14 (A644G). We hypothesized that genetic variants of this SNP can contribute to ASD development in the Kazakhstani population.

## 2. Materials and Methods

### 2.1. Subjects

Buccal swabs were collected from children with ASD, as well as from their healthy brothers and sisters, using sterile cotton-tipped applicators in individual plastic packs. Collected material was transported to the Institute of General Genetics and Cytology in a portable refrigerated container within several hours after the collection and frozen at -80°C for further molecular-genetic studies.

Collection of biomaterial was conducted exclusively on a voluntary basis after signing an informed consent by at least one of the parents. The protocol of the study was approved by the Ethics Committee of Asfendiyarov's Kazakh National Medical University (No. 57, 05.09.2017).

In addition to signing an informed consent, a detailed questionnaire and psychological testing of the children was conducted using the Childhood Autism Rating Scale (CARS) and the Modified Checklist for Autism in Toddlers (M-Chat-R). Autism severity was classified by CARS as mild/moderate (scores below 36) and severe (scores 37-60).

### 2.2. Single-Nucleotide Polymorphism (SNP) Genotyping

DNA from buccal swabs was isolated using a DNA extraction kit (AmpliSens). The DNA samples were stored at -20°C and -80°C. The PCR-RFLP assay was used for the genotyping of *MAOB* rs1799836 single nucleotide polymorphism as described earlier [1].

### 2.3. Statistical Analysis

The Hardy-Weinberg equilibrium (HWE) test for X-linked genes was used to compare the observed and expected genotype frequencies. As MAOB is located on the X chromosome, we stratified the data set on sex and analyzed males and females separately. Relative risks were estimated by odds ratios (OR) with a logistic regression 95% confidence interval. For females, the genotypes were examined using allelic and general models of inheritance; for males, only the allelic model was examined. The data are presented as median ± SD. Power was calculated using the genetic power calculator OSSE.

## 3. Results

### 3.1. General Characteristics of Patients

Starting from March 2018 to February 2019, a total of 262 ASD patients and their 126 healthy siblings were recruited for the current study. All individuals represented a Kazakhstani population. Recruitment was carried out in rehabilitation centers of Kazakhstan.

The clinical diagnosis of autism in our work was established by senior psychiatrists and assessed by the CARS for children over 3 years and the M-Chat-R for children under three years old. CARS and M-Chat-R have been used as standardized, investigator-based instruments for detection of ASDs [19, 20].

Characteristics of the ASD patients and healthy controls are summarized in Table 1. The ethnic heterogeneity of both groups was Kazakh, Russian, and other ethnicities (Armenians, Kyrgyz, Tatars, Uzbeks, Uigurs, Koreans, Kurds, Pakistanis, Turks, and Chechens for the ASD group; Armenians, Koreans, Tatars, Uzbeks, Uigurs, and Chechens for the control group). In the ASD group, 77% were males and 23% were females. In the control group, 41% were males and 59% were females.

Subjects were divided into infancy/early childhood, prepubertal, pubertal, adolescents, and adults. Data on the size of each group are represented in Table 1. The most numerous were groups under the age of 15 years (97.5% for ASD male, 98.3 for ASD female, 82.6% for control male, and 74.4% for control female) for which the M-Chat-R and the CARS test were used. The adolescent/adult subjects had a clinically confirmed, long-established diagnosis of autism.

### 3.2. Analysis of the Association of the *rs1799836* MAOB Polymorphism with the Risk of ASD in a Kazakhstani Population

The genotype distributions of the rs1799836 polymorphism were in accordance with the Hardy-Weinberg equilibrium (HWE) for both the control (*p* = 0.949) and ASD (*p* = 0.605) cases. The distribution of rs1799836 MAOB alleles and genotypes is presented in Table 2.

Since the MAOB gene is located on the chromosome X, both sexes were analyzed separately. No significant differences were observed in the allele frequencies between the ASD and controls cases (for males, *p* = 0.75, and for females, *p* = 0.76). AA genotype distribution was nonsignificantly higher in ASD female patients as compared to the controls (*p* = 0.76).

OR analysis was performed to evaluate the effect of rs1799836 on the severity of ASD. Autism severity was classified by CARS as mild/moderate (scores below 36) and severe (scores above 36). As shown in Table 3, female ASD cases with the A allele significantly tend to be severely autistic rather than mild or moderate (*p* = 0.03).

## 4. Discussion

Our family-based study failed to prove the hypothesis that rs1799836 of MAOB was associated with ASDs in the Kazakhstani population. The obtained results showed that the SNP was not significantly associated with ASD development in both males and females.

Little research has been conducted so far to study the impact of MAOB rs1799836 on ASD pathogenesis. Earlier, similar results were obtained in a study of ethnically homogenous groups of male Caucasians with autism [21]. On the contrary, MAOB G allele frequency was significantly increased in Egyptian autistic patients more than in controls (OR = 34 and 12.5 for male and female cases, respectively; *p* < 0.001) [1]. Although we cannot exclude the influence of genetic differences between populations on the results, we could also attribute the contradictory results to the smaller sample size in the aforementioned study (cases/controls, 67/42) as compared to the current study (cases/controls, 262/126), which may lead to lower power analysis.

Nevertheless, a number of studies have shown the association of rs1799836 of MAOB with other “brain diseases.” In a meta-analysis, Y. Liu *et al.* found a significant relationship between the A allele of the rs1799836 polymorphism and Parkinson's disease development [22]. The G allele of rs1799836 was identified as a risk factor for developing schizophrenia (*p* = 0.006) in a Spanish population [23] and in Han Chinese [24].

Interestingly, our analysis further demonstrated that the severity of autism significantly increased for A allele female carriers (*p* = 0.03), which can be possibly explained by the enzymatic activity of this allele. It has been earlier shown that the A allele of rs1799836 is associated with significantly lower enzyme activity in platelets as compared to the G allele [25]. The hypothesis is further supported by the fact that monoamine oxidase activity in platelets inversely correlates with personality characteristics such as impulsiveness, sensation-seeking, monotony avoidance, and to some degree aggression [26, 27]. Low platelet MAO activity has been demonstrated in alcoholics [28]. The mean MAOB activity in suicidal patients was significantly lower than in nonsuicidal depressed patients and healthy controls (*p* < 0.01) [29]. MAOB-deficient mice showed increased reactivity to stress [30]. Finally, the hypothesis that activity of neurotransmitter genes contributes to ASD severity is in line with recent findings linking MAO isoenzyme activity to ASD symptoms. Thus, it was shown that autistic males with the low-expressing 3-repeat allele of the MAOA gene had more severe characteristics of sensory behaviors, arousal regulation problems, aggression, and weaker social communication skills than males with the high activity allele [31].

In summary, the obtained results indicate that rs1799836 may not be directly related to the occurrence of ASDs but through A allele may affect the symptoms of ASDs in female patients. However, the study has several limitations. Subdivision of all individuals to male and female groups as well as to severe and mild/moderate ASD groups led to a relatively small size of the samples, so the power of these subgroup results was <80%, indicating that additional high-level studies are still needed.

The current work investigated only one polymorphism of the MAOB gene. Therefore, we cannot rule out the involvement of this gene in the ASD occurrence. To completely exclude the role of this gene in the development of ASDs, it is necessary to study other SNPs of this gene using powerful sequencing methods. The methodology of this work was limited; however, the obtained results give a good reason to increase funding for further research in this direction, which will allow improvement of the methodological level of the study.

Another possible limitation of this study is the family-based design, which may imply the “contamination” of control samples with “suspected” genes. Earlier, Evangelou et al. reported that both unrelated case-control and family-based studies gave overall similar estimates on associations in 93 subgroup analyses [32]. A key benefit of such family-based association studies is the control for confounding bias due to population stratification [33]. Sibling controls are derived from exactly the same gene pool as the cases and thus represent exactly matched controls, but they do pose other practical and statistical difficulties. Thus, it was shown that siblings are more likely to have the same genotype as the case than unrelated controls, thereby leading to some loss of statistical efficiency [34]. Considering this disadvantage of the family-based design, we agree that our findings are preliminary and need to be validated in further studies with samples of unrelated representatives of the Kazakhstani population.

## 5. Conclusion

To the best of our knowledge, this is the largest study on the association of rs1799836 of the MAOB gene with ASD risk. Even though we found no association of the rs1799836 of the MAOB gene with ASD susceptibility, we are reporting on the association of the MAOB rs1799836 marker with symptom severity in females, which implies that the SNP may be involved in pathways contributing to ASD progression in a gender-specific manner. However, further investigation is needed to validate these results. If they are replicated in future studies, the findings may be used for the development of new target therapies.

The present study is a pilot study to report on an association of the candidate genes with ASDs in the Kazakhstani population. The results represent an interesting platform for further studies on the impact of polymorphisms of neurotransmitter genes on ASDs and its characteristics.

## Figures and Tables

**Table 1:** Characteristics of ASD patients and control subjects.

Characteristic		ASD *N* (%)		Controls *N* (%)	
		Male	Female	Male	Female
Sample size		202	60	52	74

Ethnicity	Kazakh	137 (68)	39 (65)	33 (64)	62 (84)
	Russian	45 (22)	15 (25)	10 (19)	6 (8)
	Other nationality	20 (10)	6 (10)	9 (17)	6 (8)

Age (years)	Median ± SD	7.20 ± 3.69	6.73 ± 2.83	9.09 ± 6.55	9.62 ± 6.84
	Range	2-33	2-16	1-29	1-29

Periods of age	Infancy/early childhood (up to 7 years old) (%)	112 (55.4)	33 (55.0)	28 (53.8)	33 (44.6)
	Prepubertal (7 to 11 years old) (%)	57 (28.2)	21 (35.0)	5 (9.6)	13 (17.6)
	Pubertal (11 to 15 years old) (%)	28 (13.9)	5 (8.3)	10 (19.2)	9 (12.2)
	Adolescence (15 to 20 years old) (%)	4 (2.0)	1 (1.7)	6 (11.5)	14 (18.9)
	Adults (above 20 years old) (%)	1 (0.5)	0	3 (5.8)	5 (6.8)

CARS score	Median ± SD	33.43 ± 7.53	34.07 ± 7.30	15.63 ± 1.37	16.29 ± 4.13
	Range	30-52.5	30-54	15-19	15-17

**Table 2:** Genotype and allele distributions of MAOB in ASD patients and controls.

rs1799836 MAOB	ASD patients		Controls		OR	95% CI	*p* value
	No.	%	No.	%			
Male							
AA	133	66	33	63	1.11	0.59-2.06	0.75
GG	69	34	19	37	0.90	0.48-1.68	
Female							
A allele	70	58	84	57	1.14	0.70-1.86	0.76
G allele	50	42	64	43	0.88	0.54-1.43	
AA	23	37	24	32	1.30	0.64-2.64	0.76
AG	26	43	36	49	0.81	0.41-1.60	
GG	11	20	14	19	0.96	0.40-2.31	

**Table 3:** Relation between MAOB polymorphisms and severity of ASDs.

MAOB rs1799836	Severe		Mild and moderate		OR	95% CI	*p* value
	No.	%	No.	%			
Male							
A allele	48	68	72	65	1.13	0.60-1.86	0.70
G allele	23	32	39	35	0.88	0.47-1.45	
Female							
A allele	37	71	32	52	2.31	1.06-5.04	0.03
G allele	15	29	30	48	0.43	0.20-0.94	
AA	13	50	9	29	2.44	0.82–7.29	0.12
AG	11	42	14	45	0.89	0.31–2.55	
GG	2	8	8	26	0.24	0.05–1.25	

## Data Availability

The data used to support the findings of this study are available from the corresponding author upon request.
